# Evaluation of beliefs and attitudes among caregivers of child labor about mental disorders first aid and stigma

**DOI:** 10.1186/s40359-022-00792-x

**Published:** 2022-04-01

**Authors:** Laleh Ghadirian, Azadeh Sayarifard

**Affiliations:** 1 Present Address: Community Based Participatory Research Center, Iranian Institute for Reduction of High-Risk Behaviors, Tehran University of Medical Sciences, Tehran, Iran; 2 Knowledge Utilization Research Center, Tehran University of Medical Sciences, Tehran, Iran; 3 Post-Doctorate in Psychosomatic Medicine and Psychotherapy, Albert Ludwig University of Freiburg, Freiburg, Germany

**Keywords:** Belief, Attitude, Child labor, Caregiver, Mental health first aid, Stigma

## Abstract

**Background:**

The beliefs and attitudes of caregivers of working children about mental health issues and first aid and their attitudes about the stigma of mental health problems can affect their motivation to seek professional help for affected children. This study aimed to assess the mental health literacy among caregivers of child labor about first aid for mental health and their attitudes about the stigma of mental disorders.

**Methods:**

The Depression Health Literacy Questionnaire has been used in this cross-sectional study. All caregivers of working children who were willing to participate were included in the study. This group is covered by a Non-Governmental Organization (NGO) located in the 17th district of Tehran.

**Results:**

Questionnaires completed by 131 caregivers were analyzed. The average age of the participants was 32.6 (± 7.9) years. Of which 130 (99.2%) were mothers. Amongst, only 37 individuals (28.2%) were confident in their ability to help their children in case of depression symptoms. A majority of participants, 109 (83.2%), believed that asking a depressed child about suicidal ideation was harmful.

**Conclusion:**

According to the findings of this study, the literacy among caregivers of child labor about mental health first aid in our study needs to be improved. There is also a need for educational and community-based programs to reduce the stigma about mental health disorders, including depression.

## Background

The child labor phenomenon is a significant problem worldwide in the form of child labor (under the legal age for work) to help the family make a living [1]. Overall, child laborers are split into two categories: children that live in very low-income families and have to help their families. They work in production or services part-time or full-time to support their family. These children have families and a place to live and sleep and only go out on the streets for work. Another group is children who either don't have families or are drug addicts or suffering from other sorts of crises. These children go on the streets to live and work [2].

According to the International Labor Organization (ILO) statistics, 218 million children aged 5–17 years are currently employed worldwide, and 73 million of whom are in high-risk forms of child labor [3]. Statistics also show that child labor makes up about 22% of the workforce in Asia, 32% in Africa, 17% in Latin America, and 1% in the United States, Canada, Europe, and other developed countries [4]. These findings are shocking, not only because child labor is a debatable violation of children's basic rights but also because of its potential harm to their educational, physiological, and mental development [5].

A literature review shows that several studies have been conducted to assess work-related injuries, common illnesses of child labor, and their psychological injuries, including depression [6]. Studies in Iran have also shown that the prevalence of depression in street children who return home to work during the day and at home at night was 61.4%, 86.7% in girls, and 48.2% in boys [7].

The findings of these studies suggest an urgent need for further research to improve the mental health of child laborers [8].

The mental health literacy of individuals shows their knowledge about mental disorders and their understanding of the need to visit a specialist to receive necessary treatment. Therefore, an essential step in promoting the mental health of a community is to recognize the state of mental health literacy and focus on improving it if necessary[9].

A dominant solution for promoting mental health literacy is "mental health first aid" (MHFA) education. It is defined as "helping a person who has a mental health problem or is in a mental health crisis" [10–16]. MHFA includes providing support and cares to prevent harassment and interference, identifying needs and concerns, listening to individuals without judging them, helping people access information, social support and services, and protecting vulnerable individuals. MHFA is not professional counseling; instead, it is a substitute for psychological information that has been found ineffective [17].

On the other hand, stigma is a significant obstacle to seeking mental health services that can be assessed from the individual, family members, and society's viewpoint. Stigma also negatively affects the quality of life of people with mental illness [18, 19]. Therefore, beliefs and attitudes among caregivers of child labor about mental health issues and necessary first aid and mental stigma can affect their motivation to learn first aid and seek professional help for these children. Since no study has been conducted in Iran so far, and because caregivers of child labor are the closest people to them and do not have enough information in this regard, they can not provide first aid for their child if necessary. Therefore, this study was conducted to identify caregivers' level of mental health literacy about depression and the critical first aid areas that are the first step in designing and implementing the necessary interventions.

The results of this study can improve the caregivers of child labor' mental health literacy and can be a starting point for designing community-based interventions to reduce the stigma of psychiatric disorders among caregivers of child labor and hence, assist early diagnosis and help treat cases their complications. The findings of this study can also be used as baseline information to measure the effectiveness of future interventions to reduce stigma and then enhance the capacity for psychological first aid in the caregiver group.

## Methods

This study was cross-sectional. The statistical population was caregivers of under 18 years old working children who spent most of their time in low-level jobs at the workplace or on the street without adult supervision. All caregivers covered by an NGO in the 17th district of Tehran were invited to participate in the study. In this study, a caregiver is defined as a person responsible for the child's affairs, including mother, father, grandmother, etc. In this study, most caregivers (who were attended at NGO) were mothers.

Inclusion criteria included caregivers of all child labor under 18 covered by the NGO who were willing to participate in the study. Exclusion criteria were the presence of a known psychiatric illness in the child or their caregiver or the absence of a fixed caregiver. Sampling was done using the convenience method and all the caregiver, therefore including all child labor caregivers covered by the NGO.

Access to child labor and their caregivers for research is not easy, therefore the study was conducted on one NGO that covered a larger population of child labor and their caregivers, and was feasible. The researcher attended the NGO and explained the research questionnaire to two of the NGO's staff were in frequent contact with all caregivers to provide NGO's administrative services. These trained staff delivered the questionnaires to all child caregivers. The questionnaires were completed by caregivers of child labor with the guidance of the questioner (child caregivers in the organization). Verbal consent was obtained before participating in the study.

In cases when the caregiver was not literate enough to complete the questionnaire, the researcher helped participants complete the questionnaires.

Participation in the study was voluntary, stating the importance and objectives of the study for future interventions, motivated the participants, so no reward was given for participation.

The Australian mental health literacy questionnaire developed by Reavley and colleagues [20] was used, which had been previously translated and standardized by the authors of the present study. Experts approved the content validity, and the reliability was checked with the internal-class correlation coefficient (ICC) as 0.83 [21]. This questionnaire includes various areas such as recognizing the disorder, the intention to seek help and first aid, the perceived barriers, beliefs about interventions, beliefs about prevention, attitudes toward stigma, and social isolation in these study areas of beliefs and intention to seek first aid and attitudes toward stigma and social isolation were investigated.

In this questionnaire, the psychiatric disorder of the vignette is selected depending on the researcher's intention. Due to the prevalence of depression in child labor [7], a child with depression symptoms was chosen as a vignette for this study.

The Vignette was introduced as follows: Maryam/Ali is a 12-year-old who has been feeling sad and miserable for the previous weeks. She/He is continuously tired and sometimes has trouble falling sleep. She/He doesn't have appetite and has lost weight. She/He can't focus on her/his duties or studies. She/He seems agitated and aggressive. Her/His caregivers are very worried about her/him.

### Data analysis

Data analysis was performed using SPSS software. Quantity and percentage and mean and standard deviation were used to describe qualitative and quantitative variables, respectively.

## Results

None of the child labor caregivers covered by the NGO, had exclusion criteria, so all of them were included in the study, so 131 questionnaires were completed (response rate 100%). Analysis of the data showed that most of the questions were answered, and missing data was under 3%. Caregivers' mean age was 32.6 (± 7.9) years with a minimum and maximum age of 19 and 57 years, respectively. One of them was a father, and 130 were mothers. Among the participants, 114 (99.1%) persons were residents of district 18, and 1 (0.9%) was the resident of district 16 of Tehran.

Other demographic information about the participants is shown in Table 1.

**Table 1:** Characteristic of participants

	Frequency	Percent
*Education*		
Illiterate	71	54.2
Under diploma	46	35.1
Diploma	12	9.2
Missing	2	1.5
*Marriage status*		
Married	110	84
Divorced or Widowed	11	8.4
Single	10	7.6
*Occupation*		
Worker	57	43.5
Housewife	53	40.5
Vendor	19	14.5
Missing	2	1.5

In answer to the question "How confident would you be in your ability to help vignette", the responses were 37 (28.2%) sure, 43 (32.8%) unsure, 49 (37.4%) "I don't know" and 2 (1.5%) were missing data.

Participants' answers to whether first aid items can be helpful are given in Table 2.

**Table 2:** Participants` belief about first aids items

	Helpful N (%)	No helpful, no harmful N(%)	I don't know N(%)	Harmful
Listen to (his/her) problems in an understanding way	121(92.4)	6 (4.6)	3 (2.3)	0 (0)
Missing	1 (0.8)			
Make an appointment for (him/her) to see a GP or counselor if necessary	38 (29)	10 (7.6)	81 (61.8)	2 (1.5)
Ask (him/her) whether (he/she) is feeling suicidal	3 (2.3)	0 (0)	17 (13)	109 (83.2)
Missing	2 (1.5)			
Suggest (him/her) smoke cigarettes to relax	0 (0)	0 (0)	0 (0)	129 (98.5)
Missing	2 (1.5)			
Ignore (him/her) until (he/she) gets over it	34 (26)	79 (60.3)	1 (0.8)	15 (11.5)
Missing	2 (1.5)			
Keep (him/her) busy to keep (his/her) mind off problems	59 (45)	62 (47.3)	2 (1.5)	4 (3.1)
Missing	4 (3.1)			
Encourage him/her to become more physically active	60 (45.8)	60 (45.8)	7 (5.3)	2 (1.5)
Missing	2 (1.5)			

## Discussion

This study aimed to investigate the caregivers of child labor' beliefs and attitudes about first aid and the stigma of mental health problems. Most people are often aware of their physical health problems, while mental health problems are often overlooked. This issue and the stigma about mental disorders prevent people from seeking early help for their mental health problems (3). They also usually do not know how to support other persons with mental problems (7) properly. Mental Health First Aid (MHFA) training courses were prepared and implemented to improve this aspect of mental health literacy. Its five main steps include assessing the risk of suicide or harm, listening non-judgmentally, giving reassurance and information, encouraging a person to get appropriate professional help, encouraging self-help strategies [14] (Table 3).

**Table 3:** Participants` belief related to stigma

	Agree N (%)	Not agree, not disagree N (%)	Disagree N (%)
His/her problem is treatable	74 (56.5)	54 (41.2)	1 (0.8)
Missing	2 (1.5)		
His/her problem is not a real medical illness	32 (24.4)	68 (51.9)	28 (21.4)
Missing	3 (2.3)		
He/she is not dangerous to others	50 (38.2)	75 (57.3)	4 (3.1)
Missing	2 (1.5)		
It is best to avoid him/her so that you don't develop this problem yourself	11 (8.4)	65 (49.6)	52 (39.7)
Missing	3 (2.3)		
You would not tell anyone if you had a problem like him/her	15 (11.5)	74 (56.5)	40 (30.5)
Missing	2 (1.5)		

Mental health first aid emphasizes that people with mental health problems can be identified and supported similar to physical health problems by community members who can play a beneficial primary role. On the other hand, due to the high prevalence of mental health problems, community members are more likely to encounter someone experiencing a mental health crisis than a physical health crisis such as a coronary artery event, so it will be essential for them to know how to help their loved ones using first aid.

In this regard, the responsibility for caring for community members' mental health goes beyond specialized mental health services and even beyond health services in general [22, 23]. In line with the above explanation, one of the first essential questions in the first aid field for mental health is "How confident would you be in your ability to help vignette". The present study's findings showed that only 28.2% of caregivers were confident that they could help a child with a problem reported in vignette. In comparison with this result, a study conducted in adults in Tehran showed that 6% were completely and 75% somewhat confident in helping [16]. In the Yoshioka study [24], 3% of Japanese youth and in the Jorm study, 30% of Australian youth were completely confident [25].

Regarding the appropriate behavior for first aid, the majority of caregivers (92.4%) believed that listening to the child with a problem is helpful when in the study of Tehran, the majority (about 60%) considered listening to vignette as appropriate [16].

While according to studies, asking about suicidal ideation is one of the mental health first aid measures [14, 26], the majority of participants (83.2%) believed that asking this question from a person with a mental health problem would be detrimental.

This belief can be due to the stigma associated with suicide and the avoidance of any reference to it, as well as the fear of possible encouragement to attempt suicide. Only 29% of participants believed that visiting a general practitioner or counselor was helpful, which was almost similar to the findings of the Tehran survey [16]. This finding may be due to the lack of knowledge about the effectiveness of professional interventions for psychiatric disorders that in this case, providing the necessary training to increase the awareness and the ability to provide mental health first aid for the caregivers of child labor can help to raise awareness in this area.

A meta-analysis study showed that MHFA training programs could be a promising public health intervention to combat stigma and taboos for mental disorders and prevent suicide [10]. Stigma is also one of the most significant barriers to receiving specialized services by people with psychiatric disorders who refuse to accept the disease and do not seek assistance from experts in this field [27].

Based on the findings of this study, 56.5% of caregivers believed that depression, like any other disorder, can be treated; in the Ghadirian study, it was 88% in Tehran residents, and in the Sayarifard study, it was 75% in students in Tehran University of Medical Sciences [9, 20]. In the present study, similar to the findings of the Yeap study in Malaysia [28], about 24.4% of caregivers believed that depressive disorder was not a real medical illness when it was 8.6% in the Reavley study in Australia [29].

This finding is important because first aid training, correcting this wrong belief that depression is not a real illness, can affect their willingness to seek care and receive related assistance [30].

In this study, 38.2% of participants agreed that the vignette was not dangerous for others, which showed a more negative attitude compared to similar studies [9, 20, 29].

More than half of the participants responded negatively about staying away from a depressed person and not talking about this problem with others. This negative attitude can affect the request for help, initial diagnostic, and care measures as other studies have shown the relationship between stigma and help-seeking [31]. The findings of Jung's study have indicated the need to strengthen social support to promote a positive attitude towards psychological issues.

Mental health education can be useful for people at risk for mental illness and for families who are likely to influence on helping attitudes of people with mental illness [32]. On the other hand, Yoshioka's study showed that perceived stigma is significantly associated with various biogenetic, psychosocial, and personality factors [33].

Other studies have reported that despite many anti-stigma programs and extensive research in this area, it is still difficult to find effective ways to combat stigma against mental illness in the population and determine community-based interventions. More research is needed in this area [34–36].

## Limitations

Although other mental health outcomes such as anxiety, conduct problems, hyperactivity problems could be common as a consequence of child labor, this study was performed using a vignette of depression, because depression is highly prevalent in child labor, and there is not a standard questionnaire to assess all mental problems simultaneously. However, future studies are needed to investigate other common mental health disorders in child labor.

The present study was conducted only on a group of caregivers of child labor covered by an NGO in Tehran using convenience sampling and is not generalizable to all caregivers of child labor; however, since no study has been conducted on the mental health literacy in this target group in Iran so far, conducting this study is a strength to start further studies in this target group to improve their mental health and ultimately improve the mental health of child labor. Given that child labor are a disadvantaged group in society, even a slight increase in caregivers' knowledge and attitudes about MHFA can have a significant effect on the mental health of these children.

The findings of this study can be a basis for assessing the effectiveness of later training to promote depression literacy in caregivers of child labor, although selecting effective interventions in the field of depression first aid requires future studies.

## Conclusion

According to the findings of this study, the literacy among caregivers of child labor about mental health first aid in our study needs to be improved. There is also a need for educational and community-based programs to reduce the stigma about mental health disorders, including depression.

## Data Availability

The datasets during and/or analyzed during the current study are available from the corresponding author (Azadeh Sayarifard, No. 12, Nosrat Street, 16th Azar Street, Tehran, Iran, Telefax: + 98 21 66495859, Phone number: + 98 9127987191, Email: drsayarifard@gmail.com, a-sayarifard@tums.ac.ir) on reasonable request.

## Abbreviations

NGO: Non-Governmental Organization
ILO: International Labor Organization
MHFA: Mental Health First Aid
